# Allogeneic transplantation after failure of chimeric antigen receptor‐T cells and exposure to bispecific antibodies: Feasibility, safety and survival outcomes

**DOI:** 10.1111/bjh.70010

**Published:** 2025-07-22

**Authors:** Angelica Barone, Chiara De Philippis, Federico Stella, Anna Dodero, Barbara Sarina, Martina Pennisi, Armando Santoro, Carmelo Carlo‐Stella, Anna Guidetti, Stefania Bramanti, Paolo Corradini

**Affiliations:** ^1^ University of Milan Milan Italy; ^2^ Division of Hematology Fondazione IRCCS Istituto Nazionale dei Tumori Milan Italy; ^3^ Department of Oncology and Hematology Humanitas Cancer Center, IRCCS Humanitas Research Hospital Milan Italy; ^4^ Department of Biomedical Sciences Humanitas University Milan Italy

**Keywords:** immunotherapy, lymphomas, stem cell transplantation

## Abstract

Clinical outcome after chimeric antigen receptor (CAR)‐T‐cell failure in large B‐cell lymphoma (LBCL) is dismal. Allogeneic stem cell transplantation (alloSCT) represents a potentially curative salvage for relapsed/refractory LBCL, although concerns remain regarding its feasibility and safety in patients exposed to CAR‐T and bispecific antibodies. Between 2019 and 2025, 83 disease progressions were documented among 170 LBCL patients treated with CAR‐T in two academic centres; 69 (83%) started salvage treatment, the most frequent being glofitamab in 38 (55%); among those, we retrospectively analysed outcomes of 35 candidates for alloSCT consolidation. Ultimately, 13 (37%) underwent alloSCT after achieving complete (CR) or partial response. The median number of previous therapies was 5. All patients engrafted; grade III–IV acute graft‐versus‐host disease (GvHD) occurred in 8% and moderate‐to‐severe chronic GvHD in 15% of patients respectively. At 18.4‐month median follow‐up, non‐relapse mortality was 0%; all allografted patients are alive in CR; conversely, the outcome of 22 patients not proceeding to transplant was poor, with a median overall survival of 11.7 months and 13 disease‐related deaths (59%). Although in a small cohort of patients, our data highlight the potential benefit of alloSCT consolidation in selected responders to salvage regimens despite the extensive prior treatments with T‐cell redirecting therapies.

## INTRODUCTION

Anti‐CD19 chimeric antigen receptor (CAR)‐T‐cell therapy has changed the treatment paradigm for relapsed or refractory (R/R) large B‐cell lymphomas (LBCL) with durable remission rates in 30%–40% of patients both in pivotal and real‐life studies.^1^, ^2^, ^3^, ^4^, ^5^

However, approximately 60% of the patients eventually relapse,^6^, ^7^ facing a poor prognosis with a median overall survival (OS) of only 8 months.^8^, ^9^ CD3xCD20 bispecific antibodies (BsAb) have demonstrated impressive efficacy in R/R LBCL^10^, ^11^, ^12^; particularly, glofitamab monotherapy gave durable responses also in patients with prior exposure to CAR‐T cells.^13^ Such patients often present with highly refractory disease and complex clinical histories, raising concerns about the toxicities of subsequent salvage or consolidation strategies.

Allogeneic haematopoietic stem cell transplantation (alloSCT) has historically been considered a potentially curative option for those patients with R/R LBCL, provided that patients achieve a complete or at least partial remission (PR)^14^, ^15^; while alloSCT represents a potentially curative approach also in patients exposed to both bispecific antibodies and CAR‐T‐cell therapy, concerns remain regarding safety, feasibility and transplant‐related morbidity in those who had received a high number of previous therapies. Prior exposure to T‐cell redirecting therapies also raises issues on potentially high risk of infections.^16^ Previous studies highlighted the excess of transplant‐related toxicity in patients exposed to CAR‐T cells^7^ or BsAb^17^; specifically, a dismal OS of 25% at 1 year was reported in a small cohort of patients exposed to BsAb.

Herein, we report the outcomes of alloSCT in LBCL exposed to both bispecific antibodies and CAR‐T cells, with the aim of describing and evaluating the feasibility of alloSCT. Encouraging outcomes were documented in our cohort. All patients survived in remission after transplantation, highlighting the curative potential of alloSCT in this clinical setting.

## METHODS

Between February 2019 and February 2025, 170 patients affected by R/R LBCL received on‐label CAR‐T cells at Fondazione IRCCS Istituto Nazionale dei Tumori and Humanitas Cancer Center and were enrolled in the CAR‐T SIE prospective observational study (ClinicalTrials.gov ID: NCT06339255). Among 83 patients with documented disease progression, we retrospectively analysed the outcomes of 35 patients who received salvage treatment with BsAb, either within clinical trials or through expanded access programmes, and were also considered candidates for allogeneic stem cell transplantation as consolidation.

The indication for consolidation with alloSCT and eligibility for transplantation were determined at the discretion of the treating physician. Eligibility criteria included an age of less than 75 years, absence of significant cardiovascular impairment or other major comorbidities and adequate adherence to the treatment schedule. The patients considered candidates for alloSCT consolidation were defined as those for whom a donor search was initiated after the failure of CAR‐T cells. Response to treatment was assessed as per Lugano criteria.^18^

In the final analysis, we included all patients undergoing alloSCT after CAR‐T and glofitamab exposure, regardless of whether the transplant occurred immediately following glofitamab therapy or after subsequent treatments. This approach reflects the study's primary focus on evaluating the feasibility and safety of alloSCT following double exposure to T‐cell redirecting therapies. Transplantation and supportive care procedures and Statistical Methods are reported in Supporting Information.

## RESULTS

### Treatments after CAR‐T‐cell failure and characteristics of alloSCT candidates

**FIGURE 1:**
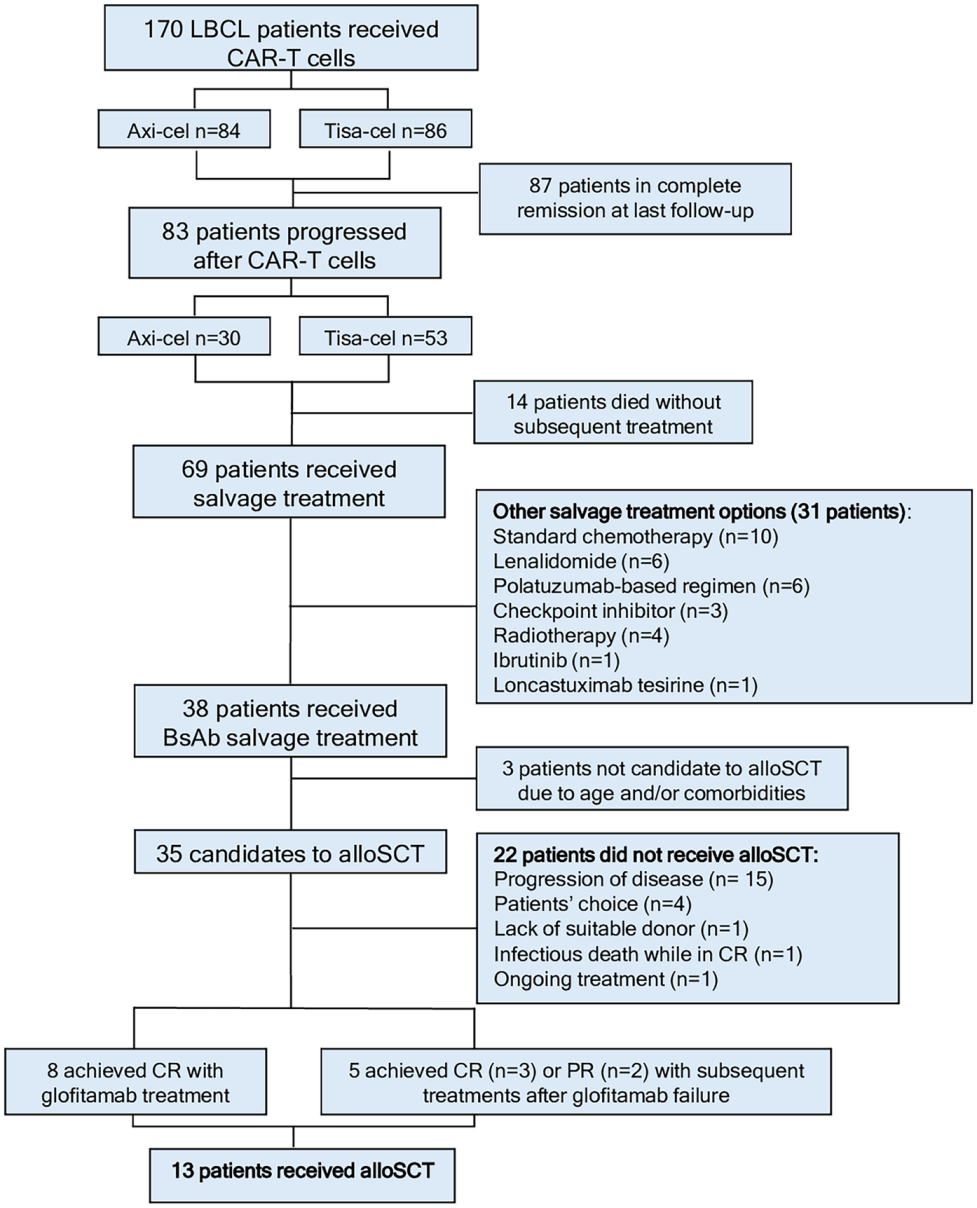
Patients' flow. alloSCT, haematopoietic allogeneic stem cell transplantation; BsAb, bispecific antibody; CAR‐T, chimeric antigen receptor T cells; CR, complete response; LBCL, large B‐cell lymphoma; PMBCL, primary mediastinal B‐cell lymphoma; PR, partial response.

From 2019 to January 2025, 170 patients with LBCL treated at Fondazione IRCCS Istituto Nazionale dei Tumori and Humanitas Cancer Center were enrolled in the prospective CAR‐T SIE study and received on‐label CAR‐T treatment. The CAR‐T product was either axicabtagene ciloleucel (axi‐cel; *n* = 84, 49%) or tisangelecleucel (tisa‐cel; *n* = 86, 51%); 83 of the 170 eventually progressed after CAR‐T‐cell therapy (axi‐cel *n* = 30, 36%; tisa‐cel *n* = 53, 64%); relapse rates were 36% and 62% for axi‐cel and tisa‐cel respectively (*p* < 0.001). Sixty‐nine patients received salvage treatment while 14 died without subsequent therapies, either due to disease progression (*n* = 12) or infections (*n* = 2) (Figure 1); the main salvage treatment option was represented by glofitamab (55%, *n* = 38; either on expanded access programme, *n* = 27, or within clinical trials Roche BP41072, BP30179 or BP43015, *n* = 11), while 31 patients received salvage treatments other than BsAb. Among 38 patients treated with BsAb, 35 were candidates for up‐front alloSCT consolidation; three were not considered for alloSCT due to pre‐existing comorbidities and inadequate performance status, at the physician's discretion. Of 31 patients receiving salvage treatments other than BsAb, nine were candidates for alloSCT consolidation and four ultimately underwent alloSCT: since we focused on the feasibility and safety of alloSCT following both CAR‐T and BsAb exposure, those were not included in the present analysis.

**FIGURE 2:**
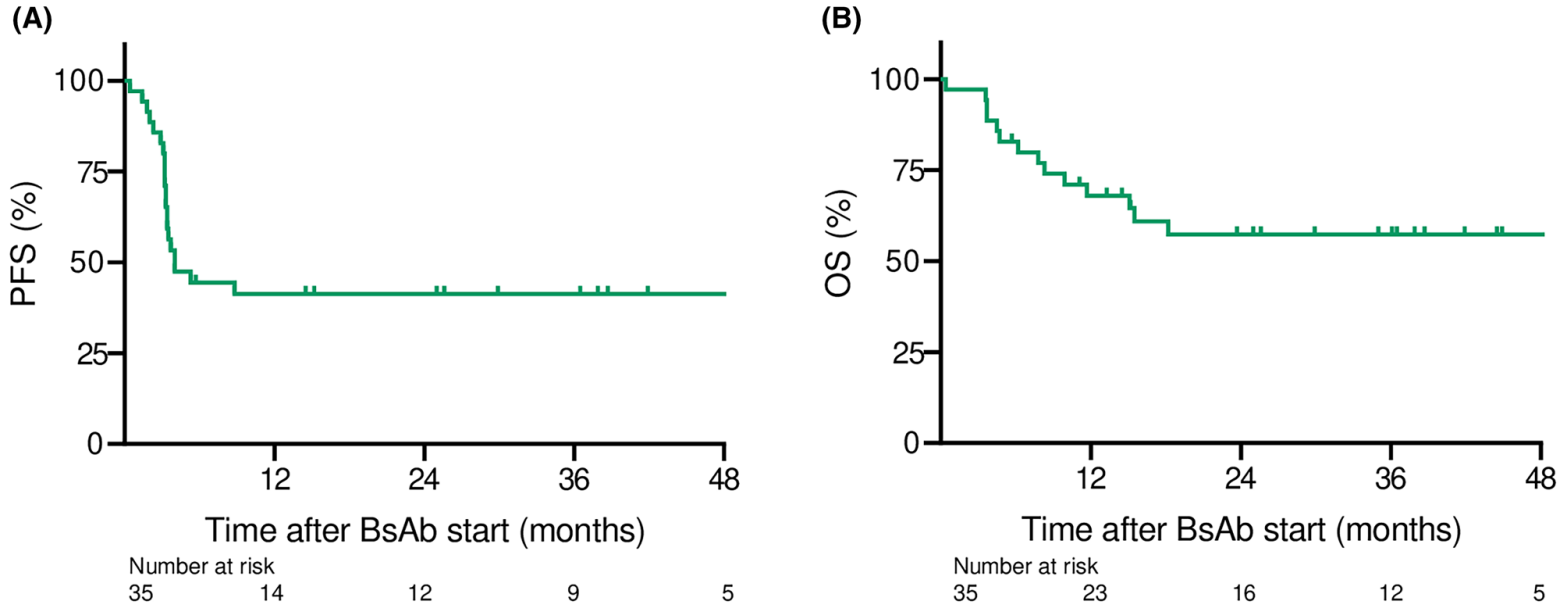
Survival outcomes in 35 intent‐to‐transplant patients receiving BsAb salvage treatment after CAR‐T failure. (A) Progression‐free survival (PFS) of 35 intent‐to‐transplant patients (median PFS: 4 months). (B) Overall survival (OS) of 35 intent‐to‐transplant patients (median OS: Not reached). BsAb, bispecific antibody; CAR‐T, chimeric antigen receptor T cells.

In 35 alloSCT candidates receiving glofitamab salvage treatment, median progression‐free survival was 4 months, while median OS was not reached (Figure 2). Overall response rate after glofitamab therapy was 63% (complete remission [CR] in 17 [49%] and partial response [PR] in 5 patients [14%] respectively); 12 patients (34%) were primary refractory. Overall, 20 patients had disease progression after glofitamab; subsequent treatments included radiotherapy (*n* = 4), polatuzumab‐vedotin‐based regimens (*n* = 3), lenalidomide (*n* = 4), loncastuximab tesirine (*n* = 2); seven patients died without receiving any further treatment. In patients either relapsed or refractory to glofitamab treatment, median time to glofitamab failure was 99 days (interquartile range [IQR]: 74–109).

Among 35 candidates for alloSCT after BsAb therapy, 13 patients (37%) proceeded to transplantation. Of note, five of them had been briefly mentioned in our previous report, although without detailed description and with a significantly shorter follow‐up.^8^ The main reason for 22 (63%) patients not receiving alloSCT was disease progression (*n* = 15; 43%), followed by patients' choice (*n* = 4; 11%), lack of suitable donors (*n* = 1; 3%), Sars‐CoV‐2‐related death (*n* = 1; 3%) or ongoing treatment with BsAb (*n* = 1; 3%). Patients who underwent transplantation were significantly younger at CAR‐T failure (median age 48 vs. 61 years, *p* = 0.006), while other baseline characteristics were not significantly different between the two groups, as outlined in Table 1. The frequency of patients who were primary refractory to CAR‐T cells or BsAb treatment was not significantly different between alloSCT and non‐alloSCT patients. We observed a trend towards longer median time to CAR‐T failure in the alloSCT group, although not statistically significant (3.9 vs. 1.8 months, respectively; *p* = 0.06).

**TABLE 1:** Baseline characteristics of patients either receiving (*n* = 13) or not (*n* = 22) alloSCT consolidation.

	alloSCT = 13	Non‐alloSCT = 22	*p*‐Value
Age at CAR‐T failure, median (IQR)	48 (33–55)	61 (54–65)	0.006
Male sex, *n* (%)	6 (46%)	18 (81%)	0.06
Histology			0.08
DLBCL, NOS	8 (61%)	20 (91%)	
DLBCL, transformed from indolent B‐NHL	4 (31%)	1 (4.5%)	
PMBCL	1 (8%)	1 (4.5%)	
CAR‐T product			>0.99
Axi‐cel	5 (38%)	9 (41%)	
Tisa‐cel	8 (62%)	13 (59%)	
Ann‐Arbor stage III–IV, *n* (%)	9 (69%)	20 (91%)	0.12
IPI ≥3, *n* (%)	5 (38%)	8 (36%)	>0.99
Bulky disease, *n* (%)	4 (31%)	5 (23%)	0.69
Median no. therapies received, *n* (range)	5 (4–9)	4 (3–10)	0.45
CAR‐haematotox high, *n* (%)	5 (38%)	13 (59%)	0.08
Primary refractory to CAR‐T treatment, *n* (%)	5 (38%)	13 (59%)	0.30
Median time to CAR‐T failure, months (IQR)	3.9 (2.1–8.4)	1.8 (1.2–3.9)	0.06
No. of cycles of glofitamab received, median (%)	7 (2–12)	5 (1–12)	0.13
Glofitamab failure, *n* (%)	5 (38%)	15 (68%)	0.08
Primary refractory to glofitamab treatment, *n* (%)	3 (23%)	9 (41%)	0.46

Abbreviations: alloSCT, haematopoietic allogeneic stem cell transplantation; axi‐cel, axicabtagene ciloleucel; B‐NHL, B‐cell non‐Hodgkin lymphoma; CAR‐T, chimeric antigen receptor T cells; DLBCL, diffuse large B‐cell lymphoma; IPI, International Prognostic Index; IQR, interquartile range; NOS, not otherwise specified; PMBCL, primary mediastinal B‐cell lymphoma; tisa‐cel, tisagenlecleucel.

### Transplanted patients

In 13 patients receiving alloSCT consolidation, median follow‐up after CAR‐T failure was 37.9 months (IQR 24.25–48.1). All of them had received glofitamab after CAR‐T failure; disease status at the time of transplantation was CR in 11 patients, achieved either with glofitamab (*n* = 8) or subsequent therapies (*n* = 3: lenalidomide, polatuzumab‐vedotin plus rituximab‐bendamustine, loncastuximab‐tesirine), and PR in two patients, achieved after radiotherapy and polatuzumab‐vedotin plus rituximab respectively.

Median number of previous therapies was 5 (range: 3–9). Five (38%) of the 13 patients had hypogammaglobulinaemia prior to transplant, defined as immunoglobulin G (IgG) count <400 mg/dL. Median time from CAR‐T failure to alloSCT was 8.9 months (range: 2.3–21.9); median time from last treatment to alloSCT was 41 days (range: 20–98), while median time from last dose of glofitamab to alloSCT was 66 days (range 34–369). Age‐adjusted haematopoietic cell transplantation–specific comorbidity index (HCT‐CI) was ≤2 in 9 (69%) patients. Eastern Cooperative Oncology Group (ECOG) Performance Status at transplantation was 0 in 12 patients and 1 in 1 patient. All patients received reduced intensity conditioning regimens; transplant conditioning intensity score^19^ was low in nine patients and intermediate in four patients. Donor source was peripheral blood stem cells (PBSC) in all patients. Donor type was matched‐related (MRD), matched‐unrelated (MUD) or haploidentical in 2 (16%), 5 (38%) and 6 (46%) patients respectively. Graft‐versus‐host disease (GvHD) prophylaxis was mainly based on post‐transplant cyclophosphamide (PTCy) plus ciclosporin (CSA) and mycophenolate mofetil (MMF) in 8 (62%) patients; 5 (38%) patients received CSA plus short course methotrexate, either alone (*n* = 1), with anti‐thymocyte globulin (ATG; *n* = 2), or with MMF (*n* = 2). Patients' and transplant's characteristics are summarized in Table 2.

**TABLE 2:** Patients' and transplant characteristics.

	Patients (*N* = 13)
Median follow‐up after HSCT, months (IQR)	18.4 (14.5–38)
Male sex, *n* (%)	6 (46%)
Disease status at HSCT, *n* (%)	
CR	11 (85%)
PR	2 (15%)
HCT‐CI score, median (*r*)	2 (0–4)
0–2, *n* pts	9 (69%)
≥3, *n* pts	4 (31%)
Donor type, *n* (%)	
MRD	2 (16%)
MUD	5 (38%)
Haplo	6 (46%)
Conditioning regimen type, *n* (%)	
FluCyTBI2Gy	5 (38.5%)
ThioFluCy	5 (38.5%)
TBF	3 (23%)
ANC engraftment, *n* (%)	13 (100%)
Median time to ANC engraftment, days (range)	19 (10–25)
PLT engraftment, *n* (%)	13 (100%)
Median time to PLT engraftment, days (range)	23 (10–45)

Abbreviations: ANC, absolute neutrophil count; CR, complete response; FluCyTBI2Gy, fludarabine + cyclophosphamide + 2 Gy, total body irradiation; HCT‐CI, haematopoietic cell transplantation–specific comorbidity index; HSCT, haematopoietic stem cell transplantation; MRD, matched related donor; MUD, matched unrelated donor; PD, progressive disease; PLT, platelets; PR, partial response; TBF, thiotepa + busulfan + fludarabine; ThioFluCy, thiotepa + fludarabine + cyclophosphamide.

All 13 patients achieved neutrophil engraftment before day 30, with a median time to engraftment of 19 days (range 10–25); median time to platelet engraftment was 23 days, occurring before day 30 in all patients but one. Full donor chimerism was documented in all patients at day +30 and +90 after infusion. Median absolute lymphocyte count at days +90, +180 and +360 after alloSCT was 0.515 × 10⁹/L (IQR: 0.410–0.635 × 10⁹/L), 0.710 × 10⁹/L (IQR: 0.610–1.510 × 10⁹/L) and 1.510 × 10⁹/L (IQR: 1.260–1.720 × 10⁹/L) cells/× 10⁹/L, respectively.

### Transplant‐related toxicities

Six (46%) patients developed acute GvHD (aGvHD), mainly grade I–II (38%), with a median time to onset of 64 days; steroids only were administered in four patients, while extracorporeal photopheresis (ECP) was added in two patients with grade II and grade III aGvHD respectively. Univariate analysis did not reveal any statistically significant differences between selected variables (namely, median time from last treatment, time from last BsAb dose and median number of BsAb cycles received) and occurrence of aGvHD (*p* > 0.5). Four patients (30%) developed chronic GvHD (Table 3): Two cases (15%) of moderate cGvHD were treated with ECP, CSA and imatinib. Median time to CSA withdrawal was 216 days (range: 154–1129); of note, one patient who developed grade III aGvHD and then chronic GvHD required prolonged low‐dose CSA and ECP treatment, discontinuing CSA at 1129 days after transplant. At last follow‐up, three patients had ongoing immunosuppressive therapy (CSA in two and imatinib in one patient with mild and moderate cGvHD respectively).

**TABLE 3:** Main transplant‐related toxicities.

	Patients (*n* = 13)
Acute GvHD, *n* patients (%)	6 (46%)
Grade I–II	5 (38%)
Grade III–IV	1 (8%)
Median time to aGVHD, days (range)	64 (27–198)
Chronic GvHD, *n* patients (%)	4 (30%)
Mild	2 (15%)
Moderate	2 (15%)
Median time to cGvHD, days (range)	159 (90–225)
Number of infectious events per patient, median (range)	1 (0–5)
Bacterial infections <100 days, *n* patients (%)	5 (39%)
*Staphylococcus epidermidis*	2 (15%)
*Pseudomonas aeruginosa*	1 (8%)
*Citrobacter kroseri*	1 (8%)
*Klebsiella pneumoniae*	1 (8%)
Viral infections, *n* patients (%)	3 (23%)
Herpes simplex virus 1	1 (8%)
Sars‐CoV‐2‐related pneumonia	2 (15%)
Number of viral reactivations per patient, median (range)	1 (0–3)
Viral reactivations, *n* patients (%)	7 (54%)
HHV6, *n* (requiring treatment)	6 (4)
CMV, *n* (requiring treatment)	6 (3)
JC virus, *n* (requiring treatment)	1 (0)
Viral reactivations >180 days, *n* patients (%)	4 (31%)
CMV	3 (23%)
EBV	1 (8%)
Fungal infections, *n* patients (%)	0

Abbreviations: aGvHD acute graft‐versus‐host disease; cGvHD chronic graft‐versus‐host disease; CMV, cytomegalovirus; EBV, Epstein–Barr virus; GvHD, graft‐versus‐host disease; HHV‐6, human herpesvirus 6; JC virus, John Cunningham virus; Sars‐CoV‐2, severe acute respiratory syndrome coronavirus 2.

The number of microbiologically documented bacterial infections after alloSCT was 0, 1 and 2 in five, five and four patients, respectively. Before day 100 after alloSCT, six bloodstream bacterial infections were documented in five patients: Median time to occurrence was 10 days (range: 4–39) and isolated microorganisms are detailed in Table 3. All events were treated with broad‐spectrum antibiotics and successfully resolved. Seven (54%) of 13 patients had at least one viral reactivation; among those, median number of viral reactivations was 2 (range: 1–3). Human Herpesvirus 6 (HHV6) and cytomegalovirus (CMV) reactivations were the most frequently detected in six patients each: association with end‐organ disease (gastrointestinal infection) was observed in two patients. Three patients (23%) had clinically meaningful CMV reactivations occurring after 180 days after transplant, successfully treated with valganciclovir, while one patient had Epstein–Barr virus (EBV) reactivation at 550 days after alloSCT and received weekly rituximab for four doses with complete resolution. No fungal infections were reported during the follow‐up period (Table 3).

Among uncommon complications after transplant, two immune‐related events were recorded in two patients: one haemolytic anaemia successfully treated with steroids at 15 months after alloSCT occurring in a patient with concomitant cGvHD, and one anti‐GM2‐ganglioside immunoglobulin M (IgM)‐positive acute demyelinating polyneuropathy diagnosed 10 months after alloSCT, successfully managed with intravenous immunoglobulins with complete neurological recovery. The same patient also had a severe, prolonged, non‐BK virus‐related haemorrhagic cystitis that took several months to resolve.

### Survival outcomes

**FIGURE 3:**
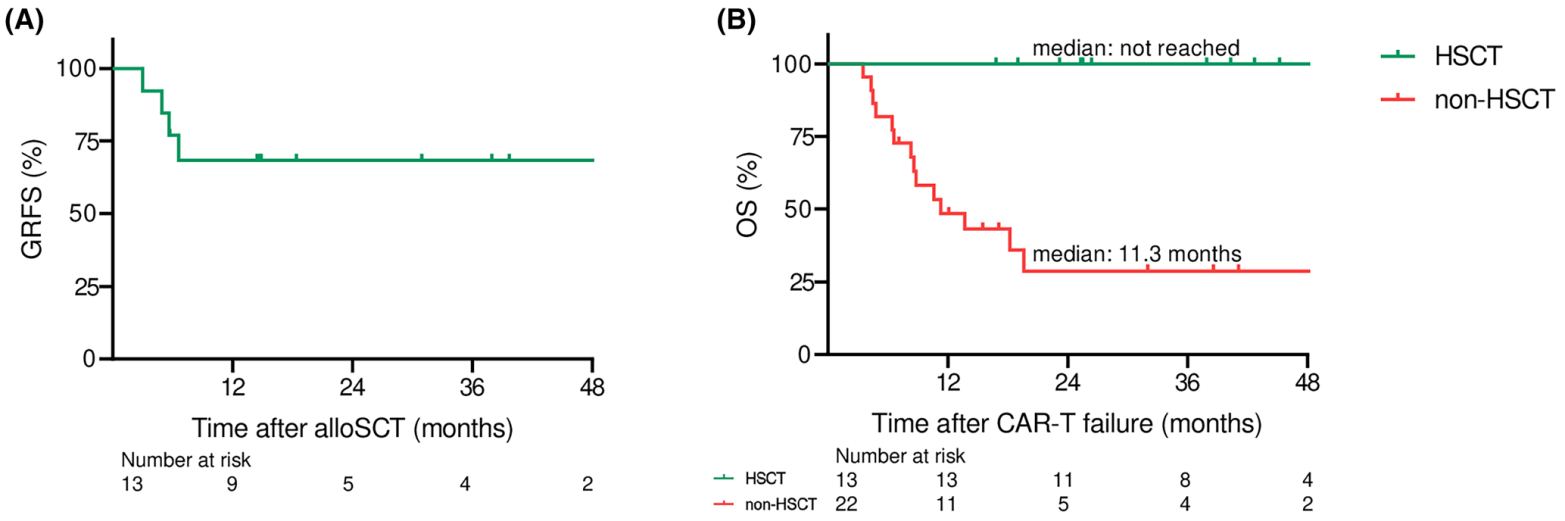
Survival outcomes of allografted and non‐allografted patients. (A) GvHD‐ and relapse‐free survival of allografted patients (median GvHD‐ and relapse‐free‐survival: not reached). (B) Overall survival (OS) of alloSCT versus non‐alloSCT patients after CAR‐T failure. Median OS not reached versus 11.3 months (log‐rank test, *p*‐value = 0.001). alloSCT, haematopoietic allogeneic stem cell transplantation; CAR‐T, chimeric antigen receptor T cells; GRFS, GvHD‐free, relapse‐free survival; GvHD, graft‐versus‐host disease; HSCT, haematopoietic allogeneic stem cell transplantation.

At last follow‐up, all 13 allografted patients were alive in complete remission, with a median follow‐up after alloSCT of 18.4 months (IQR: 14.5–38); 1‐year GvHD‐free, relapse‐free survival (GRFS) was 69% (95% confidence interval [CI]: 36.3–87.3.30) (Figure 3A). Median OS after CAR‐T failure was not reached in the alloSCT group versus 11.3 months in the non‐alloSCT group respectively (*p* = 0.001) (Figure 3B). At last follow‐up, only 8 (36%) of 22 patients were alive in the non‐alloSCT group: Five who did not receive alloSCT either due to patients' choice or lack of donors were in sustained CR after 12 cycles of glofitamab, one patient was in ongoing treatment and two were started on subsequent treatments ongoing at last follow‐up. Among 14 deaths, the main cause was disease progression (*n* = 13), except for one Sars‐CoV‐2‐related death in a patient in CR.

## DISCUSSION

Patients with LBCL that relapse or fail to respond to CAR‐T cells have a poor outcome, with a median OS after CAR‐T failure reported between 5 and 8 months across studies.^8^, ^9^ However, a significant proportion may still attain durable remissions with subsequent BsAb treatment.^10^, ^13^, ^20^ A joint position paper published in 2020 by the European Society for Blood and Marrow Transplantation (EBMT) and the Center for International Blood and Marrow Transplant Research (CIBMTR) restricts the role of alloSCT to a rescue strategy for eligible patients who respond to salvage treatments after CAR‐T failure,^21^ although this publication precedes the advent of BsAb therapies.

Nonetheless, the role of alloSCT consolidation in patients achieving CR after BsAb treatment remains controversial. Recently, an extended follow‐up of phase I/II study suggested high rates of durable responses with fixed‐duration glofitamab, with a median PFS of 31 months for patients achieving early CR at cycle 3; such outcome appeared comparable between patients with or without prior CAR‐T cell treatment, but the cohort of patients exposed to CAR‐T cells was very limited in this study.^13^ Similarly, epcoritamab and odronextamab seem to provide durable responses,^20^, ^22^ although longer follow‐up of phase I/II studies and robust real‐world data are needed before dismissing the potential role of alloSCT consolidation in this setting. Of note, our study was neither designed nor powered to detect differences in outcomes of patients either allografted or not after achieving CR with glofitamab, as most patients received alloSCT before such extended follow‐up analyses were available.

In our study, among the patients defined as intent‐to‐transplant, only a minority (37%) ultimately received alloSCT consolidation, largely because of high‐risk disease progressing after BsAb therapy as well as after all other salvage regimens. Notably, similar disease‐related baseline characteristics at CAR‐T failure indicate a comparable high‐risk disease profile across the two groups, including the percentage of patients who were refractory to CAR‐T and BsAb treatments. Unsurprisingly, a trend towards shorter time to CAR‐T failure and a higher incidence of BsAb failure was found in the non‐alloSCT group.

The heavily pretreated nature of our population and their elevated risk profile poses significant concern for transplant‐related morbidity and mortality. A recent analysis found similar safety and efficacy outcomes for patients receiving alloSCT after immunotherapy in the pre‐CAR‐T era compared to a historical cohort of patients allografted after standard chemotherapy.^23^ Consistently, our data suggest that outcomes of alloSCT after CAR‐T and BsAb exposure might be comparable to those of CAR‐T and BsAb naïve LBCL populations: with the limitation of a small sample size, the rates of engraftment (100%) and incidence of acute (46%) and chronic GvHD (30%) were in line prior studies of alloSCT for LBCL,^21^, ^24^, ^25^, ^26^, ^27^ while there were no unexpected complications with the exception of the two immune‐related events and the occurrence of one case of severe haemorrhagic cystitis. While haemorrhagic cystitis is a common complication after alloSCT, especially in the setting of haploidentical transplant,^28^ and autoimmune haemolytic anaemia is well recognized in literature and frequently arises in the context of cGvHD^29^, ^30^—which was mild in our patient—acute demyelinating polyneuropathy has been sporadically reported after alloSCT and associated with CMV reactivation and cGvHD, neither of which was present in our patient at that time.^31^, ^32^ To our knowledge, a single published study reports on alloSCT outcomes after BsAb exposure: in sharp contrast with the poor 1‐year OS of 25% in eight patients described, with five transplant‐related deaths,^17^ we observed a prolonged disease control with a 100% rate of complete remissions of lymphoma and no severe infectious complications after alloSCT. Such outcomes likely reflect patient selection: of note, patients proceeding to alloSCT were relatively young (median age: 48 years) and with a low comorbidity score (HCT‐CI ≤2 in 69% patients); periodic supplementation of intravenous immunoglobulin was administered in most patients in case of IgG count <400 mg/dL. In consideration of the high number of previous therapies received and disease status, institutional protocols called for a reduced intensity conditioning regimen,^26^ while the choice of specific regimen was largely dependent upon the centre's experience^33^, ^34^; GvHD prophylaxis was administered in accordance with recent consensus,^35^ although the increasing use of PTCy should be acknowledged.^36^, ^37^ In line with established evidence,^7^, ^14^ active refractory disease remained a major contraindication for alloSCT consolidation. Indeed, all allografted patients in our study were either in CR (85%) or PR (15%) at the time of transplantation and while this optimal disease status likely contributed to disease control, the small sample size precludes the assessment of whether CR versus PR influenced transplantation outcomes.

In conclusion, all alloSCT patients engrafted with encouraging disease control and manageable transplant‐related complications. Clearly, being deemed eligible itself and proceeding to alloSCT are positive selection factors per se; nevertheless, our data support a potential benefit of alloSCT consolidation in fit, carefully selected responders to salvage therapies. While a standard‐of‐care approach has not yet been defined for patients relapsing after CAR‐T cells, longer follow‐up and real‐world data will be key to clarifying the role of alloSCT consolidation in those achieving a CR with BsAb treatment, especially as current data on response durability remain immature and require robust confirmation in real‐life settings. Although the small size of our cohort precludes definitive conclusions about the optimal timing of alloSCT—especially in early responders who may not require consolidative transplantation,^13^ we believe that early donor identification and work‐up should be pursued in potential alloSCT candidates. Nonetheless, we encourage colleagues at other institutions to contribute patient‐level data on post‐CAR T‐cell therapy failure treated with bispecific antibodies, in order to perform a comprehensive, multicentre meta‐analysis that will more definitively characterize the efficacy and optimal sequencing of these agents in this challenging clinical setting. Finally, optimal patient selection and tailored post‐transplant monitoring remain essential in this very high‐risk population, particularly as alloSCT currently represents the only potentially curative option in the event of BsAb failure.

## AUTHOR CONTRIBUTIONS

*Conception and design*: PC, AB, FS. *Provision of study materials or patients*: AB, CDP, FS, AD, BS, AS, CC‐S, AG, MP, SB. *Collection and assembly of data*: AB. *Data analysis and interpretation*: AB, FS, PC. *Manuscript writing*: All authors. *Final approval of manuscript*: All authors. *Accountable for all aspects of the work*: All authors.

## FUNDING INFORMATION

This study is sponsored by Fondazione IRCCS Istituto Nazionale dei Tumori, Milano, Italy, Associazione Italiana contro le Leucemie‐linfomi e mieloma (AIL) Milano e Monza‐Brianza; ‘Società Italiana di Ematologia’. This work was supported by the European Union—Next Generation EU—NRRP M6C2—Investment 2.1 Enhancement and strengthening of biomedical research in the NHS (project #PNC‐E3‐2022‐23683269PNC‐HLS‐TA).

## CONFLICT OF INTEREST STATEMENT

SB—Speaker bureau: Bristol‐Myers Squibb, Gilead, Novartis; advisory board: Novartis; Travel accommodation: Novartis, Roche. PC—Speaker and/or participating in advisory board: AbbVie, ADC Therapeutics, Amgen, BeiGene, Celgene, Daiichi Sankyo, Eli Lilly, Gilead/Kite, GSK, Incyte, Janssen, Jazz Pharma, Novartis, Pfizer, Roche, Sanofi, SOBI, Takeda; honoraria for lectures: AbbVie, Amgen, Celgene, Kite/Gilead, Janssen, Novartis, Roche, Sanofi, Takeda. CC‐S—research funding from Roche, ADC Therapeutics and Sanofi; consultant/advisor for Roche, Celgene/Bristol‐Myers Squibb, ADC Therapeutics, Sanofi, Scenic Biotech; honoraria from Roche, ADC Therapeutics, Bristol‐Myers Squibb, Merck Sharp & Dohme, Janssen Oncology, AstraZeneca, Incyte, Novartis, Takeda. AS Advisory Board: Bristol‐Myers‐Squibb, Servier, Gilead, Pfizer, Eisai, Bayer, MSD (Merck Sharp & Dohme). Consultancy: Sanofi, Incyte. Speaker's Bureau: Takeda, BMS, Roche, AbbVie, Amgen, Celgene, Servier, Gilead, AstraZeneca, Pfizer, Lilly, Sandoz, Eisai, Novartis, Bayer, MSD, BeiGene. The other authors declare that they have no conflict of interest.

## ETHICS STATEMENT

The ethical committees of participating institutions approved the study.

## PATIENT CONSENT STATEMENT

All patients gave written informed consent according to the Helsinki declaration.

## Supporting information


Data S1.

## Data Availability

The dataset analysed during the current study is available from the corresponding author on reasonable request.
