# Supplementary material for: Allogeneic transplantation after failure of chimeric antigen receptor‐T cells and exposure to bispecific antibodies: Feasibility, safety and survival outcomes
Source: Br J Haematol. 2025 Jul 22;207(3):956–64. doi: 10.1111/bjh.70010 (PMC12436228; doi:10.1111/bjh.70010)
Supplement: Supplementary file 1 — Data S1. [file BJH-207-956-s001.docx]

**Allogeneic transplantation after failure of CAR-T cells and exposure to bispecific antibodies: feasibility, safety and survival outcomes**

**Supplementary Data**

**Transplantation procedures and supportive care**

Reduced-intensity conditioning (RIC) regimens used were either based on Thiotepa 5 mg/kg on day -6 and -5 plus Busulfan 3.2 mg/kg and Fludarabine 50 mg/kg through days -4 to -2 (TBF); Thiotepa 5 to 10 mg/kg on day -5 plus Cyclophosphamide 30 mg/kg and Fludarabine 30 mg/kg through days -4 and -3 (ThioFluCy); or Fludarabine 30 mg/mq through days -6 to -2 plus Cyclophosphamide 15 mg/kg on days -6 and -5 plus Total Body Irradiation (TBI) 2 Gray (FluCyTBI2Gy). Comorbidities at the time of transplantation were assessed according to the age-adjusted Hematopoietic Cell Transplantation-Comorbidity Index (HCT-CI)(1).

Post-grafting immunosuppression was based on either calcineurin inhibitor cyclosporine A (CSA) 3 mg/kg/day from day -1 plus short-course methotrexate (10 mg/mq on day +1 and 8 mg/mq on day + 3 and +6), or post-transplant cyclophosphamide (PTCy) 50 mg/kg on days +3 and +4 plus CSA and oral mycophenolate mofetil (MMF) 15 mg/kg/day from day +5; MMF was withdrawn on day +35 in absence of active GvHD. CSA dosage varied on biweekly therapeutic dose monitoring and was withdrawn between 6 to 8 months post transplantation in absence of active GvHD. Graft-versus-Host-disease (GvHD) grading was performed according to current criteria(2,3). GvHD was treated according to institutional guidelines, considering the European Society for Blood and Marrow Transplantation recommendations (4).

As per institutional protocol, all patients received prophylactic acyclovir, while letermovir was used to prevent cytomegalovirus (CMV) reactivation in 7 patients with high risk serological CMV status, given from day +1 to day 100. All patients received cotrimoxazole for Pneumocystis jirovecii prophylaxis; antifungal prophylaxis was either based on caspofungin (n=5) or triazoles (posaconazole = 6, fluconazole = 2, isavuconazole = 1). Five patients also received antibacterial prophylaxis with amoxicillin. According to institutional guidelines, CMV, Human Herpesvirus 6 (HHV6) and Epstein-Barr virus (EBV) were monitored weekly until day +100; Aspergillus antigen, Human polyomavirus 1 (JC virus) and 2 (BK virus) were monitored either weekly until day +100 or based on clinical suspicion or symptoms. Granulocyte-colony stimulating factor (G-CSF) was administered in all patients after day +5 until engraftment, defined as the first of three consecutive days with an absolute neutrophil count (ANC) ≥ 0.5 × 10⁹/L and platelet count ≥ 20 × 10⁹/L without transfusion support.

**Statistical methods**

Descriptive statistics were used to summarize patient baseline characteristics. Continuous variables were reported as medians with interquartile ranges (IQRs), while categorical variables were presented as absolute and relative frequencies. Binary associations between categorical variables were assessed using the Fisher’s exact test, while Mann-Whitney test was used to assess associations between continuous variables. Survival curves were estimated using the Kaplan–Meier method. Graft-versus-host disease–free, relapse-free survival (GRFS) was defined as the time from allogeneic stem cell transplantation to the first occurrence of grade III–IV acute GVHD, chronic GVHD requiring systemic immunosuppressive therapy, disease relapse, or death from any cause. Between-group comparisons of Kaplan–Meier curves were carried out using the log-rank test.

**References**

1. Ml S, Rf S, Bm S, Rt M, Ma P, Mb M, et al. Comorbidity-age index: a clinical measure of biologic age before allogeneic hematopoietic cell transplantation. Journal of clinical oncology : official journal of the American Society of Clinical Oncology [Internet]. 2014 Oct 10 [cited 2025 Apr 6];32(29). Available from: https://pubmed.ncbi.nlm.nih.gov/25154831/

2. Jagasia MH, Greinix HT, Arora M, Williams KM, Wolff D, Cowen EW, et al. National Institutes of Health Consensus Development Project on Criteria for Clinical Trials in Chronic Graft-versus-Host Disease: I. The 2014 Diagnosis and Staging Working Group report. Biol Blood Marrow Transplant. 2015 Mar;21(3):389-401.e1.

3. Harris AC, Young R, Devine S, Hogan WJ, Ayuk F, Bunworasate U, et al. International, Multicenter Standardization of Acute Graft-versus-Host Disease Clinical Data Collection: A Report from the Mount Sinai Acute GVHD International Consortium. Biol Blood Marrow Transplant. 2016 Jan;22(1):4–10.

4. Penack O, Marchetti M, Aljurf M, Arat M, Bonifazi F, Duarte RF, et al. Prophylaxis and management of graft-versus-host disease after stem-cell transplantation for haematological malignancies: updated consensus recommendations of the European Society for Blood and Marrow Transplantation. The Lancet Haematology. 2024 Feb;11(2):e147–59.
